# Protocol of ASPro-PD: a phase 3 trial of ambroxol to slow progression in genetically stratified Parkinson’s disease

**DOI:** 10.1007/s00415-026-13683-7

**Published:** 2026-02-18

**Authors:** Marco Toffoli, Elisa Menozzi, Mairead Cullen, Kashfia Chowdhury, Saiam Ahmed, Nick Freemantle, Joy Duffen, Richard K. Wyse, Simon R. W. Stott, Helen Matthews, David Dexter, Felicia Ikeji, Julie Moss, Paul Watts, Tom Foltynie, Karl Kieburtz, Olivier Rascol, Werner Poewe, Anthony H. V. Schapira

**Affiliations:** 1 Department of Clinical and Movement Neurosciences, UCL Queen Square Institute of Neurology, University College London, London, nW3 2Pf UK; 2 Aligning Science Across Parkinson’s (ASAP) Collaborative Research Network, Chevy Chase, MD 20815 USA; 3 UCL Comprehensive Clinical Trials Unit, Institute of Clinical Trials and Methodology, University College London, London, UK; 4 MRC Clinical Trials Unit at UCL, Institute of Clinical Trials and Methodology, University College London, London, UK; 5 Cure Parkinson’s, 120 New Cavendish Street, London, W1W 6XX UK; 6 Neurology Department, Royal Free Hospital, London, UK; 7 National Hospital for Neurology and Neurosurgery, London, UK; 8 Department of Neurology, University of Rochester Medical Center, Rochester, NY USA; 9 Departments of Clinical Pharmacology and Neurociences, Clinical Investigation Center CIC14366 and NS-Park/F-CRIN Network, Toulouse University Hospital, University of Toulouse 3 and INSERM, Toulouse, France; 10 Department of Neurology, Medical University Innsbruck, Innsbruck, Austria

**Keywords:** Parkinson, Ambroxol, clinical trial, GBA1

## Abstract

**Introduction:**

Genetic studies have identified the *GBA1* gene as a significant genetic risk factor for Parkinson’s disease (PD), with 10–15% of PD patients carrying *GBA1* variants. *GBA1* variants affect the glucocerebrosidase (GCase) enzyme, often leading to reduced GCase activity and associated altered lysosomal function, implicated in PD pathogenesis. Ambroxol, a small molecule widely used for respiratory diseases, has emerged as a potential therapeutic agent for PD, acting by increasing GCase activity. A phase 2 trial demonstrated ambroxol’s safety and efficacy in penetrating cerebrospinal fluid (CSF) and engaging with its target in PD patients, including those with *GBA1* variants.

**Methods:**

We present the protocol of the ASPro-PD trial, a phase 3, multicentre, randomised, double-blind, placebo-controlled trial, aimed at evaluating whether high-dose ambroxol improves motor and non-motor function in PD patients. The trial will enrol 330 PD patients with confirmed *GBA1* status and the primary outcome will be the combined score of parts I, II, and III of the Movement Disorders Society–Unified Parkinson’s Disease Rating Scale (MDS-UPDRS). The secondary outcomes include safety, impact on PD symptoms, and quality of life. Mechanistic and exploratory outcomes include biomarkers related to GCase activity, blood, and CSF biomarkers.

**Conclusions:**

This trial is the largest to date to study the effect of ambroxol in PD, utilise a genetically stratified PD population and will provide robust estimates of the efficacy of ambroxol in slowing PD clinical progression.

**Supplementary Information:**

The online version contains supplementary material available at 10.1007/s00415-026-13683-7.

## Introduction

According to the World Health Organisation, in 2019, 8.5 million people were living with Parkinson’s disease (PD) worldwide, and PD may have the fastest growing prevalence amongst neurodegenerative disorders [1]. Current therapy improves motor symptoms, but there is no intervention to slow progression.

Multiple associations between genetic variants and PD have been identified [2]. Variants in the *GBA1* gene are numerically the most important genetic risk factor for PD, with approximately 10–15% of individuals with PD carrying a *GBA1* variant [3–6]. *GBA1* encodes for the lysosomal hydrolase glucocerebrosidase (GCase), and *GBA1* variants are the cause of the autosomal recessive lysosomal storage disorder Gaucher disease (GD) [7]. Over 400 *GBA1* mutations have been described, and they are classified as severe or mild according to whether they cause the neuronopathic or non-neuronopathic form of GD [7]. Additional *GBA1* variants confer an increased risk of PD, but do not lead to GD and are thus referred to as risk variants [8].

*GBA1* variants can lead to reduced GCase activity and misfolded GCase proteins, with a cascade of effects that involve the autophagy–lysosomal pathway, lipid homeostasis, mitochondrial function, and endoplasmic reticulum stress [9].

Furthermore, *GBA1*/GCase pathology might be relevant even in “idiopathic” PD (i.e. no identified genetic variant), because reduction of GCase activity has been reported in individuals with PD without *GBA1* variants [10]. Given the importance of the *GBA1*/GCase pathway in PD, this has become a focus of intense research to develop interventions to slow clinical progression [11].

Evidence suggests that the small molecule ambroxol might be able to rescue GCase activity in *GBA1*-associated PD, and possibly in idiopathic PD [12–18].

Ambroxol has been widely used for the past 50 years as a mucolytic and bronchodilator agent at doses between 30 and 120 mg, with a good safety and tolerability profile [19]. Importantly, at high dosages, ambroxol can act as an inhibitory chaperone for mutant GCase, increasing its transport to the lysosome, where the acidic pH (in contrast to the cytosol) causes ambroxol to dissociate from mutant GCase, allowing it to exert its residual enzymatic function [20]. Ambroxol may also increase normal GCase transfer to the lysosome by its chaperone function and result in increased activity [20]. Ambroxol is also reported to increase the nuclear translocation of transcription factor EB, a master regulator of lysosomal biogenesis [17], which could lead to even further enhanced lysosomal function.

A phase 2 unblinded clinical trial studying the use of a high dosage of ambroxol (1260 mg/day) in PD participants, with and without *GBA1* variants, showed good penetration and target engagement in the CSF [21]. This study also indicated that at elevated dosages, ambroxol was well-tolerated in the PD population over a 6-month period, and did not adversely affect the efficacy of co-administered dopaminergic therapy [21].

Several additional trials are already underway to investigate the use of ambroxol in PD, PD-Dementia, and Dementia with Lewy bodies (DLB), as summarised in Table 1.

**Table 1:** Previous and current trials exploring the use of ambroxol for the treatment of PD and DLB

Study name	Participants	Intervention	Duration of treatment	Primary outcome	References	(Estimated) Study completion
AIM-PD	8 *GBA1*-positive PD and 9 *GBA1*-negative PD	Ambroxol 1260 mg/day	186 days	CSF biomarkers, safety and tolerability	NCT02941822	Completed
AMBITIOUS	65 GBA1-positive PD	Ambroxol 1200 mg/day vs placebo	52 weeks	MoCA	NCT05287503	15 Dec 2024
Ambroxol as a novel disease-modifying treatment for Parkinson's disease dementia	15 LBD	Ambroxol 1350 mg/day vs placebo	52 weeks	MMSE	NCT04405596	Jan 2025
High-dose ambroxol in GBA1-related Parkinson	40 *GBA1* positive PD	Ambroxol 1200 mg/day (open label)	12 months	Safety and tolerability	NCT06193421	20 Apr 2025
ANeED	172 DLB	Ambroxol 1260 mg/day vs placebo	18 months	MMSE	NCT04588285	01 Jul 2025
GREAT	80 PD	Ambroxol 1800 mg/day vs placebo	48 weeks	MDS-UPDRS3 motor scale	NCT05830396	Jul 2025
Ambroxol as a novel disease-modifying treatment for Parkinson’s disease dementia	75 mild to moderate PD with dementia	Ambroxol 1050 mg/day vs ambroxol 525 mg/day vs placebo	52 weeks	ADAS-Cog and CGIC	NCT02914366	Dec 2025
APM002	240 PD cases	Ambroxol 1200 mg vs doxycycline 100 mg/day vs ambroxol 1200 mg + doxycycline 100 mg/day vs placebo	48 weeks	MDS-UPDRS	ACTRN12623000843651	May 2026

*PD* Parkinson disease, *CSF* cerebrospinal fluid, *ADAS-Cog* Alzheimer’s disease Assessment Scale–cognitive subscale, *CGIC* Clinician’s Global Impression of Change, MoCA Montreal Cognitive Assessment, *DLB* dementia with Lewy bodies, *MMSE* Mini Mental State Examination

The primary objective of ASPro-PD (Ambroxol to Slow Progression in Parkinson Disease) is to assess whether 1260 mg/day of oral ambroxol over two years in PD is associated with an improvement in motor and non-motor function, compared to placebo. Secondary objectives include the safety and tolerability of ambroxol, and the impact on other PD features and quality of life. Mechanistic and exploratory objectives include assessment of the effect of ambroxol on biofluid biomarkers of PD and to stratify results by *GBA1* status.

## Methods

### Design and study setting

ASPro-PD is a phase 3 multi-centre UK-based, randomised, double-blind, parallel-group, placebo-controlled trial, with a 104-week blinded treatment period, followed by a 26-week open-label extension. The open-label extension, whilst maintaining the blind of the first 104 weeks, provides an opportunity both to incorporate a short 6-month period of assessment of ambroxol in the original placebo group, and enables a total period of drug exposure to satisfy registration. A panel of patients, international experts, and charity representatives informed trial design.

This clinical trial will take place at NHS hospital outpatient clinics across the UK that are experienced in managing people with PD and conducting PD clinical trials.

This trial is sponsored by University College London and run by the Comprehensive Clinical Trials Unit (UCL CCTU). The trial received research ethics committee (research ethics committee reference number 24/WA/0231) approval and is registered on www.clinicaltrials.gov (NCT05778617).

### Participants

A total of 330 participants with a diagnosis of PD and known *GBA1* status (heterozygous variant carriers or wild-type) will be recruited over a period of 2 years. Participants should be already established on a stable dose of dopaminergic treatment for at least three months prior to randomisation. They should not have disabling dyskinesia or dementia. A list of current inclusion and exclusion criteria can be found in Supplementary Table 1, and a list of prohibited medications in Supplementary Table 2. The principal action of ambroxol, preclinical data, and the results of the AiM-PD study all support the hypothesis that this drug has potential benefit for PD patients either with or without *GBA1* variants [12, 17, 18, 20, 21]. This trial aims to recruit *GBA1* positive and negative patients in equal numbers and is powered to enable a comparison in response for the groups together (primary endpoint), with a secondary outcome comparing treatment effect in the subgroups defined by *GBA1* status.

Participants meeting the inclusion and exclusion criteria will be recruited within 28 days of the screening visit. All participants will sign an informed consent form for all trial procedures and will have the option to enter a sub-study investigating cerebrospinal fluid (CSF) biomarkers, with a recruitment goal of 106 of the 330 participants.

All participants will undergo genetic testing through the PD-Frontline online study (https://pdfrontline.com) or equivalent service prior to enrolment. This involves the collection of a saliva sample from participants, extraction of DNA, sequencing of *GBA1* with Nanopore long read sequencing, Sanger confirmation of positive results [22], and LRRK2 genotyping with the KBiosciences Competitive Allele Specific PCR SNP genotyping system.

### Randomisation and blinding

Participants will be randomised 1:1 to receive either ambroxol or placebo. Randomisation will be implemented using a minimisation algorithm incorporating a random element to achieve overall balance between the randomised groups by research site, Movement Disorders Society–Unified Parkinson’s Disease Rating Scale (MDS-UPDRS) part III, *GBA1* mutation status, age, time since PD diagnosis, and levodopa equivalent dose.

The treatment allocation will be concealed from all trial personnel, including investigators, pharmacy staff, research teams at sites, participants, and the analysing trial statistician prior to assignment and during the trial, until the final participant has completed the double blind phase, and the database is locked.

Before database lock, the only data summarised by treatment allocation will be within reports to the Independent Data Monitoring Committee (IDMC), which will not be distributed outside of this committee. The unblinded statistician will be responsible for preparing these reports and will be the only person outside the IDMC who will have access to these reports. Unblinded statisticians will be excluded from any discussion on the implementation or planning of the trial.

### Intervention

Participants in the active arm will receive oral ambroxol tablets (each tablet containing 420 mg). During the titration period, they will take one tablet a day (420 mg/day) for 5 days (Days 1–5), followed by one tablet twice a day (840 mg/day) for a further 5 days (Days 6–10); thereafter, one tablet three times a day (1260 mg/day) from Day 11. Participants will continue their intake of three tablets per day (1260 mg/day) for the remainder of the blinded treatment phase, which will last a total of 104 weeks. Each stage of the dose escalation can be prolonged for up to a further 5 days if required by the development of adverse symptoms. Those participants unable to reach dose level three will discontinue IMP but will continue to contribute to trial endpoints in an intention-to-treat analysis.

Participants in the placebo arm will receive placebo tablets, which will be identical to the ambroxol tablets in size, appearance, taste, and container, and will follow an identical titration period.

At the end of the 2-year blinded treatment phase, all participants will enter the open-label extension phase, starting ambroxol one tablet a day (420 mg/day) for 5 days, followed by two tablets a day (840 mg/day) for a further 5 days; thereafter, three tablets a day (1260 mg/day) for the remainder of the open-label phase, which will last a total of 26 weeks.

### Assessment of participants and outcomes

**Fig. 1:**
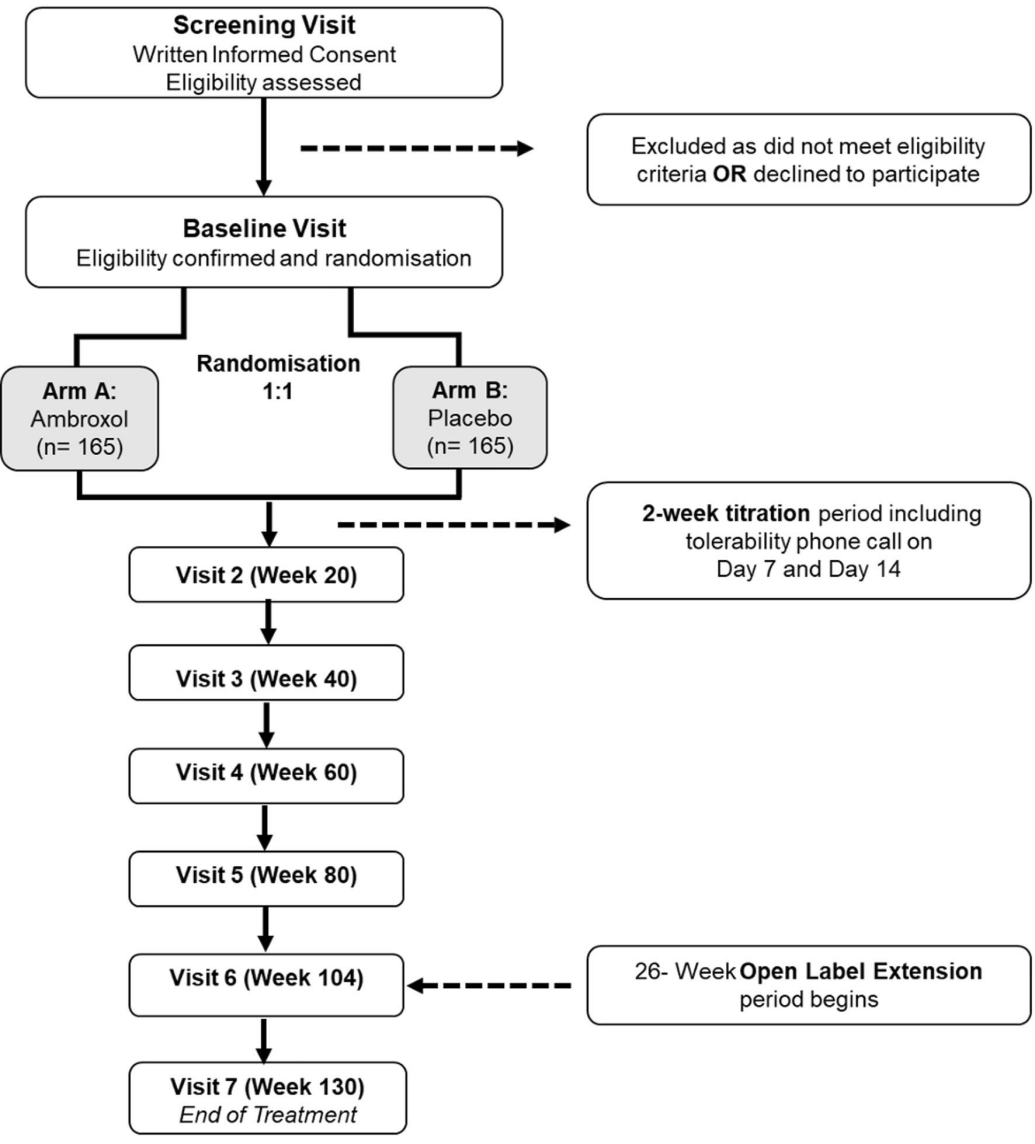
AsPRO-PD trial diagram

At screening, participants will sign an informed consent, and adherence to inclusion and exclusion criteria will be confirmed by performing vital signs, ECG, a blood test, a full neurological exam, Beck’s Depression Inventory Second Edition (BDI-II) [23] and Montreal Cognitive Assessment (MoCA) [24]. If participants meet entry criteria, they will be randomised at baseline visit, within 28 days from screening. At baseline visit, all participants will provide blood samples and will be assessed for all outcomes. Additional visits (2 to 6) will take place at 20, 40, 60, 80 and 104 weeks after randomisation, and the end of treatment visit (7) will take place 130 weeks after randomisation, i.e. at the end of the 26-week open label extension period. The assessment schedule is reported in Fig. 1.

Participants who opted to participate in the CSF sub-study will provide a CSF sample at visit 1 and at visit 6 (104 weeks).

The primary outcome is the sum score of MDS-UPDRS parts I-III (part III measured in OFF medication state) assessed at 104 weeks after randomisation. OFF medication state is defined as not having taken any PD medication for 8 h in the case of short acting agents or ≥ 24 h in the case of longer acting agents (e.g. once-daily dopamine agonists). Irreversible monoamine oxidase type B inhibitors (MAOBIs) will only be stopped the morning of the assessment and then taken after the OFF examination. It is accepted that the long half-life of MAOBIs make alternative arrangements impractical.

Different personnel will assess primary outcome measures from those documenting adverse event data to minimise risk of rater bias/inadvertent unblinding. Secondary outcomes will be assessed non-hierarchically and include the rate of change of MDS-UPDRS composite scores of parts I–III and of part III from baseline to 104 weeks, and the following outcome measures at 104 weeks: the individual scores of MDS-UPDRS parts I, II, III and IV, MoCA, Trial Making Test parts A and B [25], Hooper Visual Organization Test (VOT) [26], Parkinson’s disease 39 item Quality of Life questionnaire (PDQ-39) [27], Patient Global Impression (PGI) Severity and Change [28], EuroQol Eq-5D 5-Level Health Related Quality of Life Questionnaire [29], Non-Motor Symptoms Scale (NMSS) [30], Parkinson’s Disease Comprehensive Response (PD CORE) [31], Dresden Falls Questionnaire (DREFAQ) [32], Clinical Global Impression (CGI) [28], and change in levodopa equivalent dose. Safety and tolerability measures and time to initiation of rescue medication will also be included in secondary outcomes. Mechanistic outcomes include differences in GCase activity, GCase protein levels and alpha synuclein protein levels in blood and CSF and additionally glucosylceramide, neurofilament light chain (NFL), TAU protein and lipid profile in CSF. Further exploratory outcomes are comparison of change in MDS-UPDRS at 104 weeks from baseline by subgroup *GBA1* mutation status, analysis of *GBA1* positive and negative carrier data against placebo, change in MDS-UPDRS at 130 weeks from baseline, change in the gut microbiome composition at 104 weeks from baseline, and change in UPSIT at 104 weeks from baseline.

### Safety monitoring

At all in-person trial visits, vital signs, ECG, and blood tests will be collected. Full blood count, liver and kidney function tests, and coagulation screening will be evaluated.

Additionally, phone calls to ensure adherence and evaluate safety will be carried out at weeks 1, 2, 10, 30, 50, 70, 90, 105, 106, and 114 post-randomisation.

Adverse events (AE) will be recorded at each visit and the investigator will evaluate seriousness, severity and causality, ultimately deciding whether a participant can continue to take the treatment. All AE will be reported, disregarding their causality. The following adverse reactions are potentially related to ambroxol hydrochloride: nausea, vomiting, rash, abdominal pain, diarrhoea, anaphylactic reaction, urticaria, dyspepsia, dry mouth, numbness in throat and mouth, and changes in sense of taste.

Women of child-bearing potential will carry out a serum pregnancy test at screening, and a urine pregnancy test at each in-person visit. Adequate contraception for the duration of the study is required for such women and their male partners.

### Statistical considerations

The sample size is calculated based on estimating the average difference in MDS-UPDRS at Week 104 conditional on the baseline scores, at the 2.5% significance level in a one-sided test of superiority of ambroxol over placebo. A standard deviation of 14, a correlation of 0.7 between the outcome and baseline and 20% attrition percentage was assumed based on previous studies and data from the Exenatide-PD trial [33]. The proposed sample size has 90% power to detect the minimum important difference of 4 in MDS-UPDRS I–III at the 2.5% (one-sided) significance level.

Summary measures will be presented as means and standard deviations for normally distributed variables, medians and interquartile ranges for non-normally distributed variables, and frequencies and percentages for categorical variables.

A multilevel repeated measures linear regression model will be used to estimate the difference (with 95% confidence intervals) in MDS-UPDRS (parts I–III) scores between ambroxol and placebo, following the intention-to-treat principle. The model will include fixed effects for the intervention groups (ambroxol or placebo) and the minimisation factors (i.e. the co-variates) *GBA1* mutation status (wild-type, severe variant carrier, non-severe variant carrier), age, time since diagnosis and the levodopa equivalent dose. Baseline scores will be accounted for using a random intercept term to address clustering of repeated measurements within participants. Additionally, a random effect for site and patient effect will be included to take account of clustering. This model for the primary outcome will be extended using an interaction term to investigate whether the effect of treatment differs according to *GBA1* status, as a pre-specified subgroup analysis.

Changes to participants’ standard Parkinson medication (e.g. levodopa etc.) will be allowed during the trial. A levodopa equivalent dose (LED) will be calculated, and a causal mediation analysis may be carried out as a sensitivity analysis of the primary outcome to assess if differences in LED levels between treatment arms mediate any observed treatment effect on the primary outcome.

No formal interim analyses are planned; however, the IDMC will review trial and safety data at least annually and advise the study sponsor on the continuation or modification of the trial. Trial decisions will be taken by the Trial Management Group, with guidance and oversight from an independent committee, the Trial Steering Committee. Trial operations are delivered by the UCL Comprehensive Clinical Trials Unit.

All efforts will be made to ensure that the primary outcome data are collected for all participants. We will consider using causal mediation analysis if > 20% of participants discontinue treatment early to assess whether any potential differences in treatment adherence by arm may mediate the effect of treatment on the primary outcome. Prior to database lock, a detailed statistical analysis plan (SAP) will be produced, indicating all planned analysis and endpoints.

### Data sharing

Data will be available for sharing within 6–12 months of the trial end date. Researchers wishing to access ASPro-PD data should contact the Trial Management Group in the first instance.

## Discussion

The identification of *GBA1* variants as genetic risk factors for PD has focused attention on both GCase and lysosomal function as targets for therapeutic interventions to improve the trajectory of PD.

Ambroxol has extensive preclinical and early clinical evidence to support its trial in PD [11]. Moreover, ambroxol has an established safety profile, thanks to its long-standing use as a mucolytic agent. The results from the phase 2 study in PD extend this profile by demonstrating no adverse impact on the response to dopaminergic medications in PD [21]. This finding enabled the current study design assessing ambroxol, allowing standard of care dopaminergic medications as background therapy.

Our study includes a genetically stratified PD population and is the largest ambroxol study to date. There are several challenges to the design of disease modification trials in PD and there is no uniformly accepted model [34–36]. The design of ASPro-PD has benefitted from input and advice from several experts in the field to provide an optimised approach to recruitment and end point analysis.

We chose to recruit participants both with and without *GBA1* variants, to determine whether ambroxol has a beneficial effect in both populations, as is suggested by pre-clinical studies. Even though this will not affect the primary endpoint, we aim to recruit an equal number of *GBA1*-positive and *GBA1*-negative participants. Additional statistical analysis will be carried out to determine whether ambroxol has a differential effect between the subgroups. Moreover, *GBA1* variants were categorised as “severe” and “non-severe” and this subgroup will also be used as a minimisation factor for additional analysis.

A maximum age limit of 75 years was set to optimise the potential for follow-up during the 2.5 years of observation. Duration of disease was set at a maximum of 7 years because beyond this, the rate of decline in MDS-UPDRS slows and renders it more difficult to demonstrate a change in progression [37]. Participants will be on stable symptomatic therapy for at least 3 months prior to randomisation. They will continue therapy, but increased medication, preferably in the form of levodopa, will be allowed if considered necessary by the patient or physician. This will facilitate both recruitment and reduce dropouts.

The primary outcome measure of MDS-UPDRS I–III was supported by patient participation and confirmed following regulatory advice. The inclusion of parts I and II provides patient-driven outcomes, particularly relevant for a phase III study. Part III is measured in OFF to provide less variation and provide greater sensitivity to change [38]. A 2-year treatment period was considered the minimum to enable a meaningful difference in the primary endpoint to emerge between the treatment arms. The 6-month open label treatment period was included to both improve patient recruitment to the blinded placebo-controlled phase, and to provide the drug exposure experience required by the UK regulatory authority. The option for CSF analysis will allow establishing the relationship between changes in ambroxol levels and effect size, confirmation of target engagement with GCase protein levels, and the measurement of biomarkers including alpha-synuclein and p-tau. The biomarker outcomes have been selected to measure target engagement (blood and CSF GCase activity and protein level, CSF substrate levels) and effect on markers of neurodegeneration (CSF alpha-synuclein, TAU protein, NFL).

This study provides the opportunity to determine if ambroxol can slow the progression of symptoms in PD and whether this effect is beneficial to the whole PD population or those with *GBA1* variants.

## Supplementary Information

Below is the link to the electronic supplementary material.

Supplementary file1 (DOCX 21 KB)

## Data Availability

Data will be available for sharing after the trial end date. Researchers wishing to access ASPro-PD data should contact the Trial Management Group in the first instance.
