# Supplementary material for: Protocol of ASPro-PD: a phase 3 trial of ambroxol to slow progression in genetically stratified Parkinson’s disease
Source: J Neurol. 2026 Feb 18;273(2):151. doi: 10.1007/s00415-026-13683-7 (PMC12916515; doi:10.1007/s00415-026-13683-7)
Supplement: Supplementary file 1 — Supplementary file1 (DOCX 21 KB) [file 415_2026_13683_MOESM1_ESM.docx]

Supplementary material

**Supplementary Table 1: Inclusion and Exclusion criteria**

| **Inclusion Criteria:** |
| --- |
| 1. A diagnosis of Parkinson’s disease (in accordance with the MDS diagnostic criteria*) within 7 years of the screening visit confirmed by year of diagnosis. |
| 2. Adults aged ≥ 35 and ≤ 75 years. |
| 3. Hoehn and Yahr stage between 1-2.5, inclusive (in ON state) at screening visit. |
| 4. Known glucocerebrosidase gene (GBA1) status, mutant or wildtype, confirmed prior to the screening visit. |
| 5. On stable dopaminergic treatment for at least 3 months before enrolment. |
| 6. Able and willing to provide informed consent prior to any study related assessments and/or procedures. |
| 7. Able and willing to attend trial visits and comply with all study procedures for the duration of the trial. |
| 8. Willing and able to self-administer oral ambroxol medication or placebo. |
|  |
| **Exclusion Criteria:** |
| 1. Participation in another interventional clinical trial of an Investigational Medicinal Product (IMP) within 90 days prior to the first dose of trial treatment. |
| 2. Use of an Investigational Medicinal Product (IMP) within 90 days prior to the first dose of trial treatment. |
| 3. Participation in another clinical trial of an Investigational New Drug being tested for PD disease modifying potential within 12 months prior to the first dose of trial treatment. |
| 4. Past surgical history of deep brain stimulation. |
| 5. Use of ambroxol in the past 12 months. |
| 6. Exposure to Exenatide or other GLP-1/GIP receptor agonist within 12 months prior to the first dose in this current trial. |
| 7. Concomitant medications that are in the list of prohibited medications or that in the opinion of the Investigator would preclude participation in the study |
| 8. Confirmed dysphagia that would preclude self-administration of ambroxol. |
| 9. History of known sensitivity to the study medication, ambroxol or its excipients (microcrystalline cellulose, colloidal anhydrous silica, denatonium benzoate and magnesium stearate) that in the opinion of the investigator contraindicates their participation. |
|  |
| 10. History of known rare hereditary disorders of galactose intolerance, Lapp lactase deficiency or glucose-galactose malabsorption. |
| 11. Presence of the LRRK2 G2019S mutation (please see section 3.2.6 for further details). |
| 12. History of drug abuse or alcoholism in the opinion of the Investigator that would preclude participation in the trial. |
| 13. Pregnant or planned pregnancy during the trial and/or breastfeeding. |
| 14. Women of childbearing potential (WOCBP) and male participants with a partner of childbearing potential not willing to use highly effective contraception or abstinence (as described in section 3.2.1) for the duration of the trial treatment and for 2 weeks following the last dose of the study drug. |
| 15. Any clinically significant or unstable medical or surgical condition that in the opinion of the Investigator may; put the participant at risk when participating in the study, influence the results of the study or affect the participants ability to take part in the study, as determined by medical history, physical examinations, electrocardiogram (ECG) or laboratory tests. Such conditions may include: |
| •       Impaired renal function with creatinine clearance <50ml/min at screening visit. |
| •       Moderate or Severe hepatic impairment |
| •       A major cardiovascular event (e.g., myocardial infarction, acute coronary syndrome, decompensated congestive heart failure, pulmonary embolism, coronary revascularisation) that occurred within 6 months prior to the screening visit. |
| 16. Severe depression defined by a score >20 on the Beck Depression Inventory-II (BDI-II) at screening. |
| 17. Significant cognitive impairment defined by a score <20 on the Montreal Cognitive Assessment (MoCA) at screening. |
| 18. Use of trihexyphenidyl or benztropine within 30 days prior to the first dose of trial treatment. |
| **19. Only applicable for those patients consenting to the optional CSF sub-study:** Evidence or history of hypersensitivity to lidocaine or its derivatives. |
| **20. Only applicable for those patients consenting to the optional CSF sub-study:** Current treatment with anti-coagulants (e.g., warfarin) that might preclude safe completion of the lumbar puncture in the opinion of the Investigator. Aspirin will be permitted. |
| **21. Only applicable for those patients consenting to the optional CSF sub-study**: Significant known lower spinal malformations or other spinal abnormalities that would preclude a lumbar puncture. |

* *The MDS diagnostic criteria for probable or established PD may be used to help assist in the diagnosis*

**Supplementary Table 2: prohibited medications**

| Exenatide |
| --- |
| Dulaglutide |
| Exenatide extended release |
| Semaglutide |
| Liraglutide |
| Lixisenatide |
| Chlorpromazine |
| Metoclopramide |
| Promethazine |
| Paliperidone |
| Risperidone |
| Quetiapine |
| Clozapine |
| Trihexyphenidyl and benztropine |
| Tirzepatide* |
| Mucuna Pruriens* |
| Any other medication that in the opinion of the Investigator should preclude participation in the trial |

**this medication has been added in an amendment that is pending approval*
